# *N*,*N*′-Bis(4-bromo­benzyl­idene)ethane-1,2-diamine

**DOI:** 10.1107/S1600536808019594

**Published:** 2008-07-05

**Authors:** Hoong-Kun Fun, Valiollah Mirkhani, Reza Kia, Akbar Rostami Vartooni

**Affiliations:** a X-ray Crystallography Unit, School of Physics, Universiti Sains Malaysia, 11800 USM, Penang, Malaysia; b Chemistry Department, University of Isfahan, Isfahan, 81746-73441, Iran

## Abstract

The mol­ecule of the title Schiff base compound, C_16_H_14_Br_2_N_2_, lies across a crystallographic inversion centre and adopts an *E* configuration with respect to the azomethine C=N bond. The imino group is coplanar with the aromatic ring. Within the mol­ecule, the planar units are parallel, but extend in opposite directions from the dimethyl­ene bridge. The crystal structure is stabilized by inter­molecular C—H⋯π inter­actions and Br⋯Br [3.6307 (4) Å] short contacts.

## Related literature

For the values of bond lengths, see Allen *et al.* (1987 ▶). For related structures see, for example: Fun, Kargar & Kia (2008 ▶); Fun, Kia & Kargar (2008 ▶); Habibi *et al.* (2007 ▶); Calligaris & Randaccio, (1987 ▶). For information on Schiff base complexes and their applications, see, for example: Kia, Mirkhani, Harkema & van Hummel (2007 ▶); Kia, Mirkhani, Kalman & Deak (2007 ▶); Amirnasr *et al.* (2002 ▶); Pal *et al.* (2005 ▶); Hou *et al.* (2001 ▶); Ren *et al.* (2002 ▶).
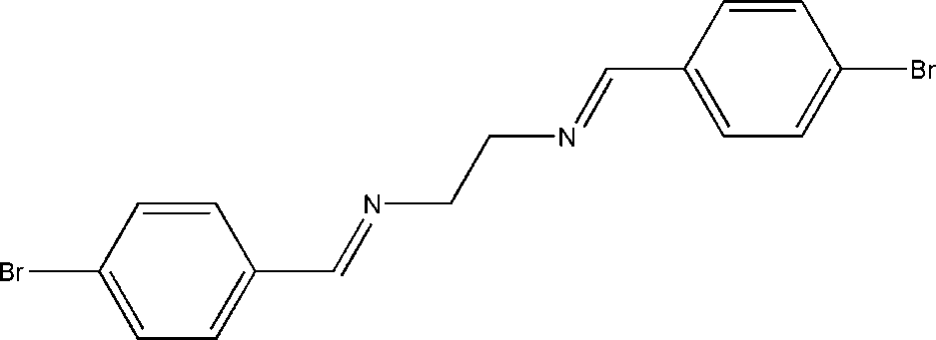


## Experimental

### 

#### Crystal data

C_16_H_14_Br_2_N_2_
*M* *_r_* = 394.11
Monoclinic, 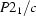

*a* = 13.8417 (5) Å
*b* = 7.4796 (3) Å
*c* = 7.1531 (3) Å
β = 95.692 (1)°
*V* = 736.91 (5) Å^3^
*Z* = 2
Mo *K*α radiation
μ = 5.49 mm^−1^
*T* = 100.0 (1) K
0.45 × 0.24 × 0.03 mm

#### Data collection

Bruker SMART APEXII CCD area-detector diffractometer
Absorption correction: multi-scan (*SADABS*; Bruker 2005 ▶) *T*_min_ = 0.189, *T*_max_ = 0.853
10096 measured reflections
2148 independent reflections
1773 reflections with *I* > 2σ(*I*)
*R*_int_ = 0.048

#### Refinement

*R*[*F* ^2^ > 2σ(*F* ^2^)] = 0.028
*wR*(*F* ^2^) = 0.069
*S* = 1.06
2148 reflections
99 parameters
H atoms treated by a mixture of independent and constrained refinement
Δρ_max_ = 0.73 e Å^−3^
Δρ_min_ = −0.45 e Å^−3^

### 

Data collection: *APEX2* (Bruker, 2005 ▶); cell refinement: *APEX2*; data reduction: *SAINT* (Bruker, 2005 ▶); program(s) used to solve structure: *SHELXTL* (Sheldrick, 2008 ▶); program(s) used to refine structure: *SHELXTL*; molecular graphics: *SHELXTL*; software used to prepare material for publication: *SHELXTL* and *PLATON* (Spek, 2003 ▶).

## Supplementary Material

Crystal structure: contains datablocks global, I. DOI: 10.1107/S1600536808019594/at2584sup1.cif

Structure factors: contains datablocks I. DOI: 10.1107/S1600536808019594/at2584Isup2.hkl

Additional supplementary materials:  crystallographic information; 3D view; checkCIF report

## Figures and Tables

**Table 1:** Hydrogen-bond geometry (Å, °)

*D*—H⋯*A*	*D*—H	H⋯*A*	*D*⋯*A*	*D*—H⋯*A*
C7—H7*A*⋯*Cg*1	0.93	2.99	3.7143 (19)	136

*Cg*1 is the centroid of the C1–C6 benzene ring.

**Fig. 1.**
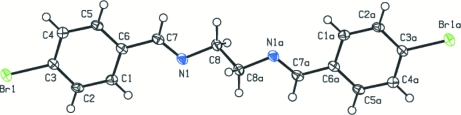
The molecular structure of (I) with atom labels and 50% probability ellipsoids for non-H atoms [symmetry code for a: -x, 0.5 + y, 0.5 - z].

**Fig. 2.**
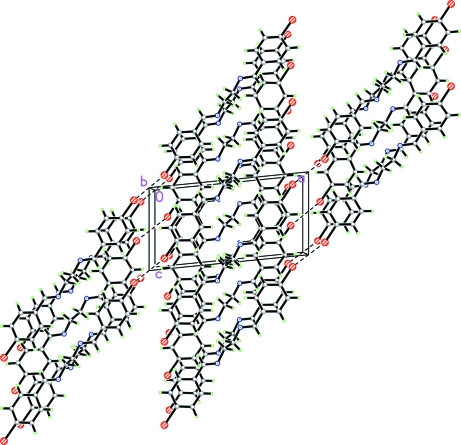
The crystal packing, showing column arrangement of the molecules along the c-axis. The Br···Br contacts are shown as dashed lines.

## supplementary crystallographic information

### Comment

Schiff bases are one of most prevalent mixed-donor ligands in the field of coordination chemistry. There has been growing interest in Schiff base ligands, mainly because of their wide application in the field of biochemistry, synthesis, and catalysis (Kia *et al.*, 2007*a*,b; Habibi *et al.*, 2007; Amirnasr *et al.*, 2002; Pal *et al.*, 2005; Hou *et al.*, 2001; Ren *et al.*, 2002). Many Schiff base complexes have been structurally characterized, but only a relatively small number of free Schiff bases have been characterized. As an extension of our work (Fun *et al.*, 2008*a*,b) on the structural characterization of Schiff base compounds, the title compound (I), (Fig. 1), is reported here.

The molecule of the title compound, (I), (Fig. 1), lies across a crystallographic inversion centre and adopts an *E* configuration with respect to the azomethine C═N bond. The bond lengths and angles are within normal ranges (Allen *et al.*,1987). The asymmetric unit of the compound is composed of one-half of the molecule. The two planar units are parallel but extend in opposite directions from the methylene bridge. The interesting feature of the structure is Br···Br*^i^* [symmetry code: (i) 2 - *x*, 1 - *y*, 1 - *z*] interactions with distance 3.6307 (4) Å. In the crystal structure, molecules (Fig. 2) are arranged into columns along the *c* axis by C—H···π interactions (Table 1).

### Experimental

The synthetic method has been described earlier (Fun *et al.*, 2008*a*,b). Single crystals suitable for *X*-ray diffraction were obtained by evaporation of an ethanol solution at room temperature.

### Refinement

H atoms bound to C8 were located from the difference Fourier map and freely refined. The rest of the hydrogen atoms were positioned geometrically with C—H = 0.93 Å and refined in riding mode with *U*_iso_ (H) = 1.2 *U*_eq_ (C).

### Crystal data

**Table:**

C_16_H_14_Br_2_N_2_	*F*_000_ = 388
*M**_r_* = 394.11	*D*_x_ = 1.776 Mg m^−^^3^
Monoclinic, *P*2_1_/*c*	Mo *K*α radiation λ = 0.71073 Å
Hall symbol: -P 2ybc	Cell parameters from 3320 reflections
*a* = 13.8417 (5) Å	θ = 3.1–31.6º
*b* = 7.4796 (3) Å	µ = 5.49 mm^−^^1^
*c* = 7.1531 (3) Å	*T* = 100.0 (1) K
β = 95.6920 (10)º	Block, colourless
*V* = 736.91 (5) Å^3^	0.45 × 0.24 × 0.03 mm
*Z* = 2	

### Data collection

**Table:**

Bruker SMART APEXII CCD area-detector diffractometer	2148 independent reflections
Radiation source: fine-focus sealed tube	1773 reflections with *I* > 2σ(*I*)
Monochromator: graphite	*R*_int_ = 0.048
*T* = 100.0(1) K	θ_max_ = 30.0º
φ and ω scans	θ_min_ = 3.0º
Absorption correction: multi-scan(SADABS; Bruker 2005)	*h* = −19→19
*T*_min_ = 0.189, *T*_max_ = 0.853	*k* = −10→9
10096 measured reflections	*l* = −10→10

### Refinement

**Table:**

Refinement on *F*^2^	Secondary atom site location: difference Fourier map
Least-squares matrix: full	Hydrogen site location: inferred from neighbouring sites
*R*[*F*^2^ > 2σ(*F*^2^)] = 0.028	H atoms treated by a mixture of independent and constrained refinement
*wR*(*F*^2^) = 0.069	*w* = 1/[σ^2^(*F*_o_^2^) + (0.0291*P*)^2^ + 0.1298*P*] where *P* = (*F*_o_^2^ + 2*F*_c_^2^)/3
*S* = 1.06	(Δ/σ)_max_ = 0.001
2148 reflections	Δρ_max_ = 0.73 e Å^−^^3^
99 parameters	Δρ_min_ = −0.45 e Å^−^^3^
Primary atom site location: structure-invariant direct methods	Extinction correction: none

### Special details

**Table:**

Experimental. The low-temperature data was collected with the Oxford Cyrosystem Cobra low-temperature attachment.
Geometry. All e.s.d.'s (except the e.s.d. in the dihedral angle between two l.s. planes) are estimated using the full covariance matrix. The cell e.s.d.'s are taken into account individually in the estimation of e.s.d.'s in distances, angles and torsion angles; correlations between e.s.d.'s in cell parameters are only used when they are defined by crystal symmetry. An approximate (isotropic) treatment of cell e.s.d.'s is used for estimating e.s.d.'s involving l.s. planes.
Refinement. Refinement of *F*^2^ against ALL reflections. The weighted *R*-factor *wR* and goodness of fit *S* are based on *F*^2^, conventional *R*-factors *R* are based on *F*, with *F* set to zero for negative *F*^2^. The threshold expression of *F*^2^ > σ(*F*^2^) is used only for calculating *R*-factors(gt) *etc*. and is not relevant to the choice of reflections for refinement. *R*-factors based on *F*^2^ are statistically about twice as large as those based on *F*, and *R*- factors based on ALL data will be even larger.

### Fractional atomic coordinates and isotropic or equivalent isotropic displacement parameters (Å^2^)

**Table:**

	*x*	*y*	*z*	*U*_iso_*/*U*_eq_	
Br1	0.901280 (16)	0.57354 (3)	−0.36941 (3)	0.02279 (8)	
N1	0.57142 (14)	0.4729 (2)	0.2936 (2)	0.0199 (4)	
C1	0.67242 (16)	0.5432 (3)	−0.0377 (3)	0.0185 (4)	
H1A	0.6068	0.5727	−0.0490	0.022*	
C2	0.72773 (16)	0.5762 (2)	−0.1849 (3)	0.0187 (4)	
H2A	0.7001	0.6316	−0.2937	0.022*	
C3	0.82508 (15)	0.5259 (3)	−0.1685 (3)	0.0172 (4)	
C4	0.86807 (16)	0.4432 (2)	−0.0085 (3)	0.0183 (4)	
H4A	0.9327	0.4073	−0.0005	0.022*	
C5	0.81247 (16)	0.4149 (2)	0.1402 (3)	0.0179 (4)	
H5A	0.8408	0.3615	0.2496	0.021*	
C6	0.71515 (16)	0.4652 (2)	0.1284 (3)	0.0169 (4)	
C7	0.65967 (17)	0.4292 (2)	0.2901 (3)	0.0185 (4)	
H7A	0.6909	0.3717	0.3944	0.022*	
C8	0.52516 (17)	0.4197 (3)	0.4604 (3)	0.0202 (4)	
H8A	0.4726 (17)	0.323 (3)	0.418 (3)	0.025 (6)*	
H8B	0.5700 (17)	0.359 (3)	0.553 (3)	0.014 (5)*	

### Atomic displacement parameters (Å^2^)

**Table:**

	*U*^11^	*U*^22^	*U*^33^	*U*^12^	*U*^13^	*U*^23^
Br1	0.02663 (14)	0.02485 (12)	0.01759 (12)	−0.00239 (9)	0.00569 (9)	0.00236 (8)
N1	0.0245 (9)	0.0218 (8)	0.0134 (8)	0.0011 (7)	0.0025 (7)	0.0012 (6)
C1	0.0196 (10)	0.0176 (10)	0.0178 (10)	0.0016 (7)	−0.0005 (8)	−0.0006 (7)
C2	0.0250 (11)	0.0172 (9)	0.0131 (9)	0.0030 (8)	−0.0015 (8)	0.0005 (7)
C3	0.0231 (11)	0.0143 (8)	0.0143 (9)	−0.0018 (8)	0.0024 (8)	−0.0009 (7)
C4	0.0193 (10)	0.0164 (9)	0.0188 (9)	−0.0014 (7)	0.0006 (8)	−0.0018 (7)
C5	0.0239 (11)	0.0148 (9)	0.0144 (9)	−0.0008 (8)	−0.0008 (8)	0.0002 (7)
C6	0.0249 (11)	0.0122 (8)	0.0135 (9)	−0.0011 (7)	0.0013 (8)	−0.0026 (6)
C7	0.0265 (11)	0.0155 (9)	0.0132 (9)	−0.0012 (8)	0.0002 (8)	−0.0010 (7)
C8	0.0231 (11)	0.0215 (10)	0.0167 (10)	0.0002 (8)	0.0050 (8)	0.0021 (8)

### Geometric parameters (Å, °)

**Table:**

Br1—C3	1.8987 (19)	C4—C5	1.389 (3)
N1—C7	1.267 (3)	C4—H4A	0.9300
N1—C8	1.464 (3)	C5—C6	1.393 (3)
C1—C2	1.384 (3)	C5—H5A	0.9300
C1—C6	1.400 (3)	C6—C7	1.475 (3)
C1—H1A	0.9300	C7—H7A	0.9300
C2—C3	1.393 (3)	C8—C8^i^	1.526 (4)
C2—H2A	0.9300	C8—H8A	1.05 (2)
C3—C4	1.383 (3)	C8—H8B	0.97 (2)
			
C7—N1—C8	116.51 (18)	C4—C5—H5A	119.4
C2—C1—C6	120.0 (2)	C6—C5—H5A	119.4
C2—C1—H1A	120.0	C5—C6—C1	119.23 (18)
C6—C1—H1A	120.0	C5—C6—C7	118.59 (18)
C1—C2—C3	119.43 (19)	C1—C6—C7	122.15 (19)
C1—C2—H2A	120.3	N1—C7—C6	123.10 (19)
C3—C2—H2A	120.3	N1—C7—H7A	118.5
C4—C3—C2	121.58 (18)	C6—C7—H7A	118.5
C4—C3—Br1	119.00 (15)	N1—C8—C8^i^	109.9 (2)
C2—C3—Br1	119.42 (15)	N1—C8—H8A	107.5 (13)
C3—C4—C5	118.43 (19)	C8^i^—C8—H8A	108.8 (13)
C3—C4—H4A	120.8	N1—C8—H8B	112.2 (12)
C5—C4—H4A	120.8	C8^i^—C8—H8B	113.6 (13)
C4—C5—C6	121.22 (19)	H8A—C8—H8B	104.4 (18)
			
C6—C1—C2—C3	2.1 (3)	C4—C5—C6—C7	179.13 (17)
C1—C2—C3—C4	0.1 (3)	C2—C1—C6—C5	−2.6 (3)
C1—C2—C3—Br1	−179.35 (14)	C2—C1—C6—C7	179.26 (17)
C2—C3—C4—C5	−1.7 (3)	C8—N1—C7—C6	176.96 (18)
Br1—C3—C4—C5	177.70 (14)	C5—C6—C7—N1	178.68 (18)
C3—C4—C5—C6	1.2 (3)	C1—C6—C7—N1	−3.2 (3)
C4—C5—C6—C1	1.0 (3)	C7—N1—C8—C8^i^	129.5 (3)

Symmetry codes: (i) −*x*+1, −*y*+1, −*z*+1.

### Hydrogen-bond geometry (Å, °)

**Table:**

*D*—H···*A*	*D*—H	H···*A*	*D*···*A*	*D*—H···*A*
C7—H7A···Cg1	0.93	2.99	3.7143 (19)	136
